# Rehabilitation of Severe-to-Profound Hearing Loss in Adults in Sweden

**DOI:** 10.3390/audiolres12040044

**Published:** 2022-08-20

**Authors:** Christian Löfvenberg, Satu Turunen-Taheri, Per-Inge Carlsson, Åsa Skagerstrand

**Affiliations:** 1 Faculty of Medicine and Health, Örebro University, 70281 Örebro, Sweden; 2 Department of Otorhinolaryngology, Central Hospital, 65230 Karlstad, Sweden; 3 Department of Audiology and Neurotology, Karolinska University Hospital, 17164 Stockholm, Sweden; 4 Department of CLINTEC, Division of Audiology, Karolinska Institutet, 17177 Stockholm, Sweden

**Keywords:** audiological rehabilitation, severe hearing loss, profound hearing loss, sensorineural hearing loss, mixed hearing loss, cochlear implant, hearing aid

## Abstract

Severe-to-profound hearing loss (STPHL) can affect a person negatively in many ways. Audiological rehabilitation is important for these patients. Patients receiving cochlear implants make up less than 10% of this group but have been studied extensively. In 2005, a national registry for adult patients with STPHL was introduced in Sweden. Its purpose was to evaluate and improve rehabilitation for all patients with STPHL. Data from the Swedish registry for adult patients with STPHL were used to evaluate variables affecting the audiological rehabilitation. Previous published data from the registry were reviewed, and new data from the follow-up questionnaire were presented. More than 90% of patients rehabilitated with hearing aids experienced a good or very good benefit of audiological rehabilitation. Tinnitus and vertigo affected quality of life negatively and were reported by many patients with STPHL (41% and 31%) at follow-up. To maintain the high number of patients who find audiological rehabilitation beneficial, individualized treatment plans and timely re-evaluations are crucial. Tinnitus and vertigo need to be addressed repeatedly in the rehabilitation process.

## 1. Introduction

Hearing loss in general and severe-to-profound hearing loss (STPHL), in particular, can result in major implications for a patient. It is well established that STPHL can lead to reduced quality of life (QoL), isolation, dependence, lack of energy, and frustration [1,2]. Furthermore, it can also lead to higher levels of anxiety and depression [3,4]. The grading system of hearing loss (HL) has recently been revised by the World Health Organization (WHO), and the following definitions are used: mild (20–34 dB), moderate (35–49 dB), moderately severe (50–64 dB), severe (65–79 dB), profound (80–94 dB), or complete HL (≥95 dB). HL in general affects many people globally, and the WHO estimates that 1.57 billion people suffer from it [5]. The WHO estimates the global prevalence of STPHL as 0.3–0.7% [5]. A previous definition of STPHL, which is still commonly used in Europe, defines it as a hearing level ≥70 dB in the better ear [6]. In Sweden, the prevalence of patients with a hearing level ≥70 dB is 0.28% [7].

STPHL can be divided into purely sensorineural HL (SNHL), where the origin of the HL can be found in the cochlea or the vestibulocochlear nerve, and mixed HL (MHL), which is a combination of SNHL and conductive HL caused by damage to the outer and/or middle ear.

Johnson points out that patients with hearing loss of a 60-dB air-conducted hearing level or less and with purely conductive hearing loss often are successfully rehabilitated with conventional HA. However, patients with MHL may have significant problems utilizing amplification [8]. This demonstrates that patients with a more pronounced MHL distinguishes from the group of patients with a lesser degree of purely conductive hearing loss and intact inner ear function.

In clinical practice, it is also well known that patients with conductive or mixed HL in general have less pronounced sensorineural damage, and they are often more successfully rehabilitated technically, especially with hearing aids, and hence could be believed to have a less marked negative impact on daily life.

Technical rehabilitation is essential for patients with STPHL, and bilateral fitting of hearing aids (HAs), bilateral cochlear implants (CIs), or bimodal hearing are beneficial [1,9,10]. However, according to recently released guidelines for the treatment of patients with STPHL, technical rehabilitation alone is not sufficient to meet the needs of this vulnerable group of patients [11]. Extended audiological rehabilitation, also called multi-professional rehabilitation, is important and can comprise meetings with several specialists, such as medical audiologists, technical audiologists, speech therapists, psychologists, and physiotherapists.

Evaluation of interventions for medical conditions is very important. Treatments administered to patients with STPHL have been extensively studied; however, they have also been overlooked. For instance, treatment with CIs and surgery for far-advanced otosclerosis have been studied thoroughly, and many studies have shown a positive impact on the QoL [12,13]. However, only a small proportion (5–10%) of patients with STPHL receive CIs [14,15]. Most patients in this group are fitted with HAs, and a small proportion receive no technical rehabilitation [16]. Only few studies have specifically evaluated the rehabilitation process of patients with STPHL who did not receive CIs.

A national registry for adults with STPHL was launched in 2005 to evaluate the rehabilitation provided to individuals with STPHL in Sweden [17]. The registry collected data on the interventions provided and their outcomes. The inclusion criteria included a hearing level of ≥70 dB or speech perception of 50% or less in the better ear. In 2015, the registry replaced the initial general questionnaire with a new baseline and follow-up questionnaire. Several previous studies have presented results from the general and the baseline questionnaires, but this is the first time that data from follow-up questionnaires have been reported [3,15,16,18]. Since the registry contains questions concerning many different rehabilitation modalities, not only specific technical rehabilitation, such as CI, this study attempted to investigate the entire population with STPHL in Sweden, regardless of intervention.

The general aim of this study was to present a summary of previously published data on audiological rehabilitation from the Swedish registry for adult patients with STPHL and present new data from the follow-up questionnaires.

The specific aims were as follows:

(1) To present data on variables that could influence the outcomes of audiological rehabilitation.
(2) To evaluate the influence of the type of HL (SNHL/MHL) and speech recognition on the outcomes of audiological rehabilitation.
(3) To compare the outcomes of audiological rehabilitation over time between HA and CI users.

## 2. Materials and Methods

### 2.1. The Swedish Registry for Adult Patients with STPHL

All data used in this study were collected from the Swedish registry for adult patients with STPHL, henceforth referred to as the registry [17]. The inclusion criteria according to the registry comprised having a bilateral HL with a pure tone average across the frequencies 0.5, 1, 2 and, 4 kHz (PTA4) ≥ 70 dB hearing level or having speech recognition in a quiet environment ≤50% in the better ear. The inclusion criteria in the registry correspond to the CI criteria in Sweden.

A previous study by our group gathered information through a set of additional questions on the presence and severity of tinnitus and vertigo, together with the Hospital Anxiety and Depression Scale (HADS), from 1274 patients in the registry [3]. The HADS is a validated 14-item self-assessment instrument consisting of one 7-item subscale for the detection of anxiety and a 7-item subscale for the detection of depression [19,20]. The validated Swedish version of this instrument was used in this study [21]. In accordance with most other studies, 8 points were used as thresholds for anxiety and depression [19]. A higher score suggested a higher level of psychological distress.

Health care registries that aim to evaluate health care quality in Sweden are called ‘national quality registries’. Swedish law requires the validation of these registries. The Swedish registry for adult patients with STPHL is one of these ‘national quality registries’. The data were validated by manual random sampling and subsequent controls at the three registering clinics. Additionally, a structured validation was performed during 2021/2022 for the audiometric data, but the results have not yet been published.

### 2.2. General and Baseline Questionnaire

The registry started in 2005 using a general questionnaire. The content of this questionnaire has been described in detail in previous studies [3,16]. In 2015, a new version, called the baseline questionnaire, and a follow-up questionnaire were introduced. The baseline questionnaire was completed during an appointment at a hearing clinic when a new rehabilitation intervention was initiated. Twelve months after the baseline, a follow-up questionnaire was sent by mail to the registered person. When completed, the patient was to return the questionnaire to the hearing clinic. Both questionnaires consisted of 2 versions: one version for the medical professional and one for the patient.

The baseline questionnaire contained data on age, sex, PTA4, speech recognition, type of HL (SNHL or MHL), communication method, the use of HAs or CI, extended audiological rehabilitation, employment, and sick leave. The unaided speech recognition test was performed in a quiet environment with a fixed speech level; the scoring was presented as correctly identified speech stimuli. Aided speech recognition in the soundfield was optional to register. In addition, the patients were asked to grade the benefits of audiological rehabilitation on a four-level scale, ranging from no benefit to very good benefit, and to estimate the degree to which HL affects their daily life (estimation scale (ES)). In the ES, the patient graded to what extent HL affects daily life on a scale ranging from 0 to 100. Similarly to previous studies based on the data from the registry, a threshold of ≥70 was used in this study to mark the point where HL caused a major negative effect on daily life [3]. Extended audiological rehabilitation was defined as rehabilitation with at least three different specialists (audiologists, technicians, psychologists, speech therapists, and physiotherapists) or participation in group rehabilitation.

Furthermore, the baseline questionnaire contained questions regarding tinnitus and vertigo. The questions were as follows: *Do you have tinnitus/vertigo?* The alternatives were either yes or no. The subsequent questions were *If yes, does tinnitus/vertigo affect your daily life?* The response options were yes, always; yes, often; yes, sometimes; no, never.

Information on reasons why the patient did not receive a CI were registered. The reasons were divided into the following groups: medical (medical conditions making CI impossible); hearing-related (the need for hearing rehabilitation is met by existing HA); patient-related (the patient has declined CI); communication (the patient uses sign language); unknown. Missing answers to this question were interpreted as unknown reasons in the baseline and follow-up questionnaires. Initiated CI investigations were also registered. The reasons why patients did not receive a CI were analyzed in the baseline and follow-up questionnaires between 2015 and 2021.

### 2.3. Follow-Up Questionnaire

The follow-up questionnaire contained questions from the baseline questionnaire and a supplemental question, where the patient was asked to rate how the influence of HL on their daily life had changed compared to one year earlier. The rehabilitation interventions introduced during the past year were registered by a professional. Rehabilitation interventions were divided into five categories: medical interventions (e.g., otosclerosis surgery), technical interventions (e.g., HA/CI), pedagogical interventions (e.g., communication strategies), psychosocial interventions (e.g., motivational support), and other interventions (e.g., referral to physiotherapists). The present study focused on the degree to which patients had received extended audiological rehabilitation and the reported benefit of hearing health care. The development of these two parameters over time was studied among HA and CI users.

Furthermore, the registry has a separate questionnaire evaluating CI surgery and the outcomes of rehabilitation with CI. This questionnaire was introduced in 2021 and is based on the International Outcome Inventory for Hearing Aids (IOI-HA) to enable comparisons. All questions were constructed according to the IOI-HA [22]. However, there were still too few registrations in the CI questionnaire to perform suitable analyses and draw robust conclusions.

In this study, data were collected from all questionnaires of the registry (general, baseline, and follow-up), except for the recently introduced CI-questionnaire. With this model, we were able to utilize all collected data in the registry, including new and previously published data.

### 2.4. Statistical Analysis

Previously published data (Table 1 and Table 2) are presented as proportions (%). Complete statistical methods and results are described in the referenced articles.

In Table 3 demographics are presented as numbers and proportions (%). Data in Table 4, Table 5, Table 6 and Table 7 are presented as total numbers in the analyses and proportions (%). Logistic regression models were performed (Table 4, Table 5 and Table 6) to evaluate the association between extended audiological rehabilitation, the benefit of audiological rehabilitation and, the benefit of hearing aids/cochlear implant with the studied variables, the type of HL, and unaided speech recognition. The variables were dichotomized. Both crude and adjusted models for potential confounding factors, such as sex, age class, and education level, were fitted. Data on education level were not available in the follow-up questionnaire. The measure of association was assessed using odds ratios (ORs) with 95% confidence intervals. Categorical data were analyzed using the chi-square test. The significance level was set at *p* < 0.01. Overall, there were small differences between the crude and adjusted ORs, and therefore, only adjusted ORs are presented in the tables. In Table 7, the two largest proportions in each subgroup are marked in bold font. All statistical analyses were performed using the IBM SPSS Statistics version 26 (Chicago, IL, USA).

## 3. Results

### 3.1. General and Baseline Questionnaire

In Table 1 and Table 2, a summary of previously published data are presented with appropriate references. Table 1 describes the use of HAs, CIs, and extended audiological rehabilitation according to sex, age at registration, education level, degree of HL (dB), and deaf blindness [16,18]. A higher proportion of women underwent extended audiological rehabilitation. In patients aged ≥81 years, the frequency of HA use and the completion of extended audiological rehabilitation was the highest and lowest, respectively, among all age groups. Regarding CI recipients, there were differences in education levels. CI use was the most common in the group in which the highest level of education was a college education. The degree of HL affected the HA and CI variables, with CI being the most common in patients with a PTA4 > 100 dB. This group had the lowest proportion of HA users.

**Table 1:** Summary of published results from *The Swedish registry for adult patients with STPHL.* The proportion of hearing aid users (HA), cochlear implant users (CI), and extended audiological rehabilitation distributed by sex, age at registration, education level, degree of hearing loss (dB), and dual sensory loss.

		HA (%)	CI (%)	Extended Audiological Rehabilitation (%)	Reference
***Total***		87	10	38	[16]
***Sex***	Female	86	12	43	[16]
	Male	88	9	34	
***Age at registration, years***	19–40	78	11	44	[16]
	41–60	83	17	53	
	61–80	88	12	44	
	≥81	92	4	20	
***Education level***	Elementary school	90	7	34	[16]
	Secondary school	86	13	41	
	Vocational school	81	6	45	
	Folk high school	90	9	42	
	College	84	17	48	
	Other education	86	11	34	
***Degree of hearing loss, dB***	>100	67	24	43	[16]
	91–100	86	16	53	
	81–90	91	11	41	
	70–80	94	2	29	
***Deaf blindness***	Dual sensory loss	89	8	32	[23]
	STPHL	86	12	40	

STPHL, severe-to-profound hearing loss.

Table 2 presents data on HADS anxiety, HADS depression, and ES variables, as well as their association with the time of onset of HL, tinnitus, vertigo, Cis, and deaf blindness. Tinnitus, vertigo, and dual sensory loss had considerable negative impacts on all these variables, while CI had a positive impact in the ES [3,23].

**Table 2:** Summary of published results from *The Swedish registry for adult patients with STPHL.* The proportions of patients with higher levels of anxiety, depression, and ES distributed by time of onset, tinnitus, vertigo, audiological rehabilitation including cochlear implants, and deaf blindness.

		HADS Anxiety ≥ 8 (%)	HADS Depression ≥ 8 (%)	ES > 70 (%)	Reference
***Onset of HL***	<3 years	31	22	40	[3]
	≥3 years	30	24	25	
***Tinnitus***	Often, always	54	37	55	[3]
	Sometimes, never	26	17	38	
***Vertigo***	Often, always	59	45	55	[3]
	Sometimes, never	33	21	42	
***Cochlear implant***	Yes	37	18	30	[3]
	No	30	23	40	
***Deaf blindness***	Dual sensory loss	41	34	50	[23]
	STPHL	29	19	36	

HADS, Hospital Anxiety and Depression Scale; ES, Estimation Scale; STPHL, severe-to-profound hearing loss; HL, hearing loss; MHL, mixed hearing loss; SNHL, sensorineural hearing loss.

Table 3 shows the demographic properties of the included patients divided into the two subgroups, MHL and SNHL. In total, 664 patients (16%) had MHL. More women had MHL than men. Patients with MHL were slightly older and had a lower level of education.

**Table 3:** Demographics in *The Swedish registry for adult patients with STPHL*; patients with mixed hearing loss (MHL) and sensorineural hearing loss (SNHL), respectively. Total number of patients, *n* = 4114.

	*MHL* *n* = 664	*SNHL* *n* = 3450
**Sex, *n*, %**		
Men	299 (45%)	1769 (51%)
Women	365 (55%)	1681 (49%)
**Age classes (years), *n*, %**		
19–40	17 (3%)	348 (10%)
41–60	86 (13%)	657 (19%)
61–80	349 (53%)	1417 (41%)
81–100	212 (32%)	1028 (30%)
**Education, *n*, %**		
Elementary school	294 (45%)	1296 (38%)
Training school	34 (5%)	201 (6%)
High school	150 (23%)	1035 (30%)
Other education	84 (13%)	363 (11%)
University	97 (15%)	545 (16%)

Table 4 shows that CI was more common in patients with SNHL than in those with MHL (12% vs. 4%). Of patients with MHL, 95% were fitted with HAs, whereas the corresponding number in the group with SNHL was 89%. Patients with unaided speech recognition of ≤50% were less likely to be fitted with HAs but had more often received a CI and attended extended audiological rehabilitation to a higher degree than did patients with unaided speech recognition >50 dB.

**Table 4:** The proportions (%) and adjusted odds ratios (OR) with 95% confidence intervals for patients with hearing aids (HA), cochlear implant (CI), and extended audiological rehabilitation, comparing mixed hearing loss vs. sensorineural hearing loss and unaided speech recognition ≤50% vs. >50%, respectively.

	HA	CI	Extended Rehabilitation
***MHL, %***	95	4	45
***SNHL, %***	89	12	45
***Adjusted OR (95% confidence interval), p-value***	2.23 ^a^ (1.52–3.27) *p* < 0.001	0.32 ^b^ (0.21–0.49) *p* < 0.001	1.02 ^c^ (0.85–1.23) ns
***Speech recognition* *≤*** ***50%, (%)***	92	16	58
***Speech recognition >50%, (%)***	97	2	47
***Adjusted OR (95% confidence interval), p-value***	0.40 ^d^ (0.26–0.61) *p* < 0.001	7.41 ^e^ (4.69–11.69) *p* < 0.001	1.55 ^f^ (1.27–1.90) *p* < 0.001

Total number of patients in analyses: ^a^ 3991, ^b^ 3825, ^c^ 3567, ^d^ 1878, ^e^ 1791, ^f^ 1631; OR adjusted for sex, age class, and education.

In Table 5, no significant differences were shown in either HADS anxiety or HADS depression when comparing the type of hearing loss and level of speech recognition. Forty-three percent (43%) of the patients with speech recognition of ≤50% had an ES value over 70 compared with 38% in patients with speech recognition of >50%.

**Table 5:** The proportions (%) and adjusted odds ratios (OR) with 95% confidence intervals for patients with higher levels of anxiety, depression, and ES, comparing mixed hearing loss vs. sensorineural hearing loss and unaided speech recognition of ≤50% vs. >50%, respectively.

	HADS Anxiety ≥ 8	HADS Depression ≥ 8	ES ≥ 70
***MHL, %***	32	25	42
***SNHL, %***	31	22	39
***Adjusted OR (95% (confidence interval), p-value***	1.18 ^a^ (0.82–1.71) *p* = ns	1.26 ^b^ (0.84–1.88) *p* = ns	1.05 ^c^ (0.88–1.27) *p* = ns
***Speech recognition* *≤*** ***50%, (%)***	29	22	43
***Speech recognition >50%, (%)***	27	19	38
***Adjusted OR (95% (confidence interval), p-value***	1.13 ^d^ (0.75–1.70) *p* = ns	1.26 ^e^ (0.81–1.98) *p* = ns	1.23 ^f^ (1.02–1.50) *p* = ns

The total number of patients in analysis: ^a^ 1113, ^b^ 1116, ^c^ 3470, ^d^ 495, ^e^ 499, ^f^ 1751. OR adjusted for sex, age class, and education.

### 3.2. Follow-Up Questionnaire

Table 6 presents the proportion of patients who, at follow-up, had completed extended audiological rehabilitation and the proportion of patients who experienced good or very good benefit of audiological rehabilitation one year after a new rehabilitation intervention was initiated. A higher proportion of patients with SNHL and those with speech perception ≤50% attended extended audiological rehabilitation but experienced less benefit from technical audiological rehabilitation (HA/CI) than did patients with MHL and those with a speech perception >50%. Overall, all groups experienced a good or very good benefit of rehabilitation to a high degree (>90%). The presence of troublesome tinnitus and vertigo (often or always) was analyzed in the follow-up questionnaire, and the proportions were 41% and 31%, respectively.

**Table 6:** The proportions (%) and adjusted odds ratios (OR) with 95% confidence intervals for patients with extended audiological rehabilitation, benefit of audiological rehabilitation, and benefit of hearing aids (HA)/cochlear implant (CI), distributed by type of hearing loss and unaided speech recognition level.

	Extended Audiological Rehabilitation	Good/Very Good Benefit of Rehabilitation	Good/Very Good Benefit of HA/CI
**STPHL, total, %**	52 ^a^	93 ^b^	90 ^c^
***MHL, %***	43	97	96
***SNHL, %***	53	93	90
***Adjusted OR (95% confidence interval), p-value***	0.67 (0.54–0.83) *p* < 0.001	2.78 (1.40–5.52) *p* < 0.003	2.58 (1.48–5.50) *p* < 0.001
**Speech recognition, total, %**	50 ^d^	94 ^e^	91 ^f^
***Speech recognition ≤50%, (%)***	53	93	87
***Speech recognition >50%, (%)***	45	95	96
***Adjusted OR (95% confidence interval), p-value***	1.48 (1.23–1.78) *p* < 0.001	0.64 (0.41–0.98) *p* = ns	0.28 (0.18–0.43) *p* < 0.001

Total number of patients in analysis: ^a^ 1570, ^b^ 2176, ^c^ 2097, ^d^ 1012, ^e^ 1507, ^f^ 1453. OR adjusted for sex and age classes.

**Figure 1:**
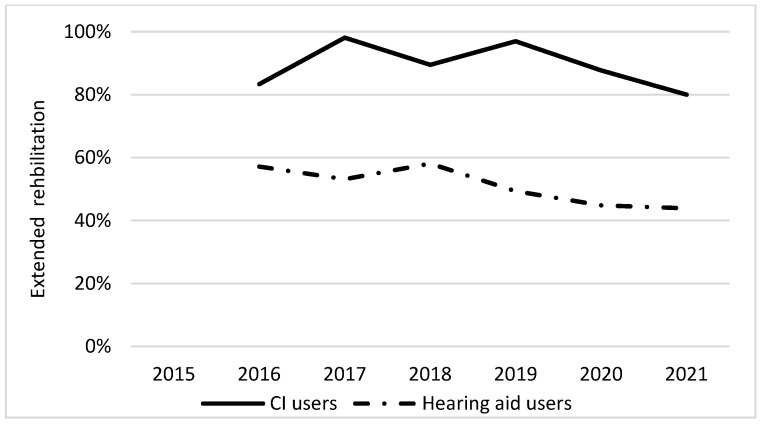
Proportion of cochlear implant (CI) users and hearing aid users who, at follow-up, had received extended audiological rehabilitation between 2016–2021.

**Figure 2:**
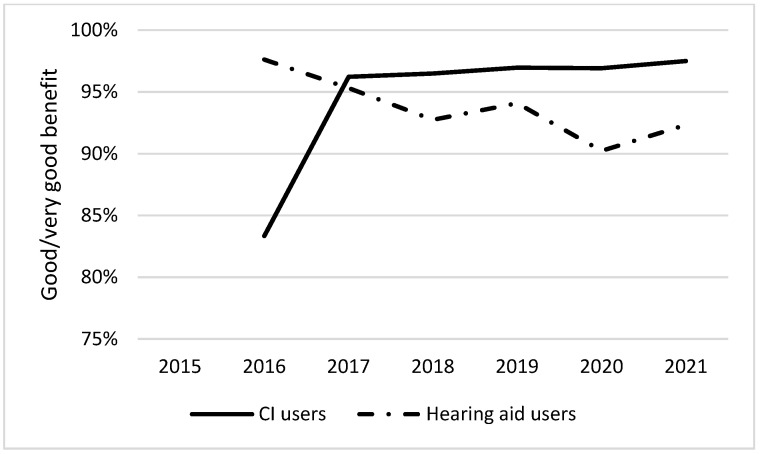
Proportion of cochlear implant (CI) users and hearing aid users who, at follow-up, experienced good or very good benefit of audiological rehabilitation between 2016–2021.

Figure 1 and Figure 2 show the differences in the proportion of patients attending extended audiological rehabilitation and the differences in the patient-perceived benefit of audiological rehabilitation between CI users and HA users over time. A higher proportion of CI users attended extended audiological rehabilitation than did HA users, and CI users also benefitted from audiological rehabilitation to a greater extent. From 2018 to 2020, there was a decline in the proportion of patients completing extended rehabilitation among HA users.

Table 7 presents the reasons why patients with STPHL were not rehabilitated with CI. In the baseline questionnaire, the most common reason was hearing-related (36.1%), whereas ‘unknown reason’ was registered in 27.5% of the patients. The proportion of unknown reasons for not being rehabilitated with CI was considerably lower in the follow-up questionnaires compared to the baseline questionnaires (28% vs. 13%). In the follow-up questionnaires, hearing-related reasons were the most common reason, followed by patient-related reasons (Table 7). The subgroup analysis revealed that 56% of patients with MHL had hearing-related reasons for not being rehabilitated with CI, compared to 37% of patients with SNHL.

**Table 7:** Reasons why patients with severe-to-profound hearing loss were not rehabilitated with cochlear implants. Data from baseline and follow-up questionnaires.

	Medical	Hearing	Patient	Communi-cation	CI Invest Start	Unknown
***Baseline***	(%)	(%)	(%)	(%)	(%)	(%)
**STPHL, total ^a^**	44	**36**	17	2	14	**28**
***Follow-up***	(%)	(%)	(%)	(%)	(%)	(%)
**STPHL, total ^b^**	5	**39**	**23**	3	17	13
**MHL** **^c^**	8	**56**	**15**	0	8	13
**SNHL** **^d^**	5	**37**	**25**	4	18	12
**Speech recognition ≤** **50%** **^e^**	5	**38**	**23**	2	19	13
**Speech recognition >50%** **^f^**	5	**42**	**23**	3	14	15

Total numbers: ^a^ 4940, ^b^ 3153, ^c^ 420, ^d^ 2621, ^e^ 1214 and, ^f^ 828. The two largest proportions in each subgroup are marked in bold font. STPHL, severe-to-profound hearing loss; MHL, mixed hearing loss; SNHL, sensorineural hearing loss.

## 4. Discussion

It is very important to evaluate rehabilitation interventions in audiological rehabilitation, particularly in patients with STPHL. Since rehabilitation should be individualized, the evaluation is essential for all patients. Patients with STPHL rehabilitated with CI experienced a greater benefit from rehabilitation and completed the extended (multiprofessional) audiological rehabilitation more often than patients without CI. However, more than 90% of patients rehabilitated with HA also experienced good or very good benefit of audiological rehabilitation (Figure 1 and Figure 2).

The present study focused on the follow-up questionnaire and measured different outcome variables one year after a new rehabilitation intervention was introduced.

### 4.1. General and Baseline Questionnaire

Previous studies on registry data have shown that the use of HA, CI, and extended audiological rehabilitation differs between groups of patients with STPHL [16,18]. Most results were expected, but the influence of the degree of HL and unaided speech recognition was especially notable. As the HL progressed, CI use became more prevalent and reached its peak in the group of patients with PTA4 >100 dB (23.6%). Consequently, and unsurprisingly, HA use showed reciprocal progression, and the largest proportion of HA users was found among patients with less pronounced HL (70–80 dB) (Table 1) [16]. Furthermore, CIs were more common in patients with SNHL than in patients with MHL. Patients with unaided speech recognition ≤50% were a little less likely to be fitted with HAs. This is explained by the fact that they received CI to a higher degree (in line with Swedish CI criteria) than did those with speech recognition >50 dB.

Tinnitus, vertigo, and deaf blindness are known to have a considerable negative impact on anxiety, depression, and daily life, while CI had a positive impact in the ES (Table 2) [3,23]. This positive impact in the ES corroborates findings from other studies investigating CI and QoL [1]. The degree of HL and unaided speech recognition had a minor impact in this context. The fact that deaf blindness has a major impact on daily life is well known [23] and will not be discussed further in this article.

In a study of patients with STPHL, Turunen-Taheri et al. revealed variations in the benefits of audiological rehabilitation associated with different audiological interventions and professions. Patients participating in group rehabilitation and visiting a hearing rehabilitation educator experienced the greatest benefits of rehabilitation. That study showed no significant differences between visits to other professionals and the benefits of rehabilitation [18].

### 4.2. Follow-Up Questionnaire

In a previous study based on the general questionnaire, troublesome tinnitus and vertigo (often or always) were shown to affect 38% and 28%, respectively, of patients with STPHL [3]. Since tinnitus and vertigo affected the QoL measures in that study (HADS-anxiety, HADS-depression, and ES), these entities are important to consider at follow-up. In this study, troublesome tinnitus and vertigo impacted 41% and 31% of patients, respectively, at follow-up. Tinnitus and vertigo remained at similar levels as in the general questionnaire, highlighting the need to monitor these parameters repeatedly in the rehabilitation process.

It is also important to remember that STPHL rehabilitation is not a static process. Re-evaluation must be performed at regular intervals. Turton et al. emphasized the need for a personalized treatment plan that must be adaptive and is updated continuously [11].

Table 6 shows that patients with SNHL received extended rehabilitation more frequently than did patients with MHL. This is likely explained by the fact that CI is more common among patients with SNHL (12%) than in those with MHL (4%), and as Figure 1 clearly depicts, almost all CI recipients receive extended rehabilitation. The decline in patients receiving extended rehabilitation among HA users from 2020 to 2021 might be explained by the restricted access to healthcare imposed by the coronavirus pandemic.

The major prerequisite for a patient to receive a CI in Sweden is speech recognition of <50% when tested in a soundfield, wearing optimized HAs. Hearing-related reasons (aided speech recognition >50%) were the most common reasons for not receiving a CI at follow-up, meaning that the hearing needs were met by HAs (Table 7). Furthermore, comparing patients with MHL and those with SNHL, a considerably greater proportion of patients in the MHL group reported hearing-related reasons for not receiving a CI (55% vs. 37%), indicating that HA fitting is more successful in patients with MHL. An additional explanation for the increased proportion of hearing-related reasons was that more patients had performed aided speech recognition tests in a soundfield at follow-up compared with baseline. In particular, patients with MHL did not meet the CI criterion with well-adapted HAs and were therefore included in the category of hearing-related reasons. We were pleased to see that the proportion of unknown reasons dropped from 27.5% at baseline to 12.8% at follow-up, indicating that CI had been considered as an option for more patients. Naturally, not all patients with STPHL will need a CI or be eligible for one, but the reason for this should be clear to both medical professionals and patients.

Only small differences were observed between CI users and HA users with respect to the patient-reported benefits of hearing health care over time. Most patients (>90%) experienced good or very good benefits of hearing health care at follow-up (Figure 2). It is important to maintain these high numbers, and this reiterates the need for individualized treatment plans and re-evaluations on a timely basis. To achieve this goal, the registry board provides annual statistical updates to audiologists from all regions in Sweden that participate in the registry.

### 4.3. Strengths and Limitations

In Sweden, the prevalence of adult patients with STPHL is 0.28%, which corresponds to 22,000 patients [7]. A limitation of studies based on the national registry for adults with STPHL is that different numbers of patients have been included in the published studies. This is because data were extracted at different periods. Missing data are an inherent problem in registry-based studies, which is also the case in this study. This means that percentages cannot always be compared directly, and each analysis must be interpreted independently. The audiological departments in all regions (except one) participated in the register. Hence, the population coverage is high. Although the number of patients varied in different analyses, the total numbers were generally high, and we are confident that the studied populations were reasonably representative of the total population of patients with STPHL in Sweden. When analyzing data on large samples, one must bear in mind that seemingly small differences between groups can still render statistical significance. Furthermore, statistical significance does not always indicate a clinical significance. For this reason, we only considered differences between groups at a significance level of *p* < 0.01. Additionally, in all types of questionnaires, it can be difficult for patients to choose between two options in a question; therefore, data are often dichotomized. This reduces the amount of information, but it is worth sacrificing some information for the sake of clarity. Robust and clear results are important when attempting to improve the rehabilitation process, which is the main purpose of the Swedish national registry for adults with STPHL. Based on a directive from the Swedish association of local authorities and regions, members from the registry board have contributed to the establishment of a national guideline for standardized care for patients with STPHL that has been approved and will be implemented later this year.

## Data Availability

The data in this study were used under license from the Swedish registry for adult patients with STPHL. Data from the registry are not publicly available, but access can be granted upon reasonable request.
